# Impact of Prematurity and Neonatal Complications on the Development of Dyslexia

**DOI:** 10.1002/jdn.70021

**Published:** 2025-05-04

**Authors:** Miguel López‐Zamora, Nadia Porcar‐Gozalbo, Isabel López‐Chicheri, Alejandro Cano‐Villagrasa

**Affiliations:** ^1^ Universidad de Málaga Málaga Spain; ^2^ Universidad Internacional de Valencia Valencia Spain; ^3^ Universidad Católica de Murcia Murcia Spain

**Keywords:** dyslexia, language impairment, neonatal complications, premature birth, reading difficulties

## Abstract

Prematurity has been linked to an increased risk of neurodevelopmental disorders, including dyslexia, due to neonatal complications that can impact brain maturation, such as intraventricular haemorrhage, periventricular leukomalacia and respiratory distress syndrome. This study examines the relationship between prematurity, neonatal conditions and dyslexia, using a sample of 120 participants divided into four groups: preterm children with dyslexia (G‐PREDIX), preterm children without dyslexia (G‐PREMA), full‐term children with dyslexia (G‐DISLX) and full‐term children without dyslexia (G‐NODISLX). Key neonatal variables such as gestational age, birth weight, APGAR scores, neonatal complications and NICU admission were analysed in relation to reading performance, assessed through standardized reading tests. Using multiple linear regression models, the study explored whether these early‐life factors predict reading difficulties and dyslexia risk. The results indicate that neonatal complications and prematurity alone do not significantly predict dyslexia diagnosis, but a negative trend was observed between intraventricular haemorrhage and periventricular leukomalacia and reading comprehension and word decoding performance. These findings suggest that prematurity, in the absence of other risk factors, does not necessarily result in dyslexia, but when combined with specific neonatal conditions, it may increase the severity of reading difficulties. These results emphasize the importance of early assessment and targeted intervention programs to support the reading development of at‐risk preterm children, particularly those with a history of neonatal complications.

## Graphical abstract

This study examines the impact of prematurity and neonatal complications on dyslexia. While prematurity alone does not predict dyslexia, specific neonatal conditions, such as intraventricular haemorrhage and periventricular leukomalacia, may exacerbate reading difficulties. These findings highlight the importance of early assessment and intervention for at‐risk preterm children.

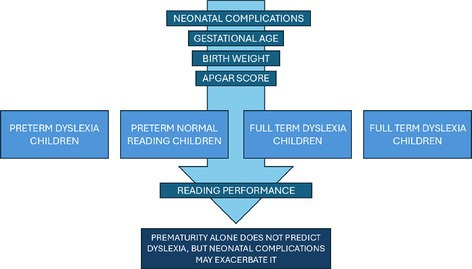

## 1 Introduction

Prematurity, defined by the World Health Organization (WHO) as birth occurring before 37 weeks of gestation, poses a critical public health issue due to its high incidence and the severe short‐ and long‐term consequences on the neurological and physical development of neonates (World Health Organization 2016). In the literature, prematurity has been associated with a higher incidence of neonatal medical complications, including respiratory distress syndrome, intraventricular haemorrhage, periventricular leukomalacia and neonatal sepsis, all of which have a potential impact on the development of the central nervous system (Saigal and Doyle 2008).

Exposure to adverse intrauterine factors and the aftermath of physiological immaturity compromise fundamental biological processes of the developing brain, particularly the myelination of white matter, neurogenesis and synaptogenesis (Volpe 2009). These processes are essential for establishing neural networks involved in higher cognitive functions, including language and reading skills. Various studies have suggested that prematurity is a risk factor for neurodevelopmental disorders, including cerebral palsy, autism spectrum disorder and, specifically, developmental dyslexia (Aarnoudse‐Moens et al. 2009).

Brain development in preterm neonates is subject to multiple disruptions during a critical period of growth, in which the connections between the cerebral cortex and subcortical areas, such as the basal ganglia and thalamus, are still forming (Back and Rosenberg 2014). Some studies have proposed that this immature process could affect neuronal pathways involved in phonological processing, a key component in language decoding and reading (Pugh et al. 2001).

One of the most studied mechanisms in the pathogenesis of brain damage in preterm infants is the dysfunction in the periventricular white matter, a region particularly vulnerable to hypoxia‐ischemia (Back 2015). This damage can alter oligodendrogenesis, interfering with the normal development of white matter tracts such as the arcuate fasciculus, involved in integrating phonological, orthographic and semantic information, and the superior longitudinal fasciculus, related to linguistic and reading abilities (Frye et al. 2010; Vandermosten et al. 2016). Diffusion MRI studies have reported reductions in fractional anisotropy in these tracts, suggesting alterations in the organization and microstructural integrity of white matter in preterm neonates compared to full‐term neonates (Beaulieu et al. 2005; Dubois et al. 2008).

The myelination process in preterm neonates is also altered, adversely affecting the speed and efficiency of neuronal transmission in brain pathways crucial for reading. Studies have demonstrated that incomplete myelination of the arcuate fasciculus, an important pathway connecting Broca's and Wernicke's areas, underlies deficits in audio‐verbal integration, crucial for phonological encoding (Yeatman et al. 2012). These findings are consistent with the reading difficulties observed in developmental dyslexia, where similar alterations in white matter are identified (Anderson and Doyle 2008; Rimrodt et al. 2010).

Furthermore, preterm neonates exhibit a high incidence of periventricular leukomalacia and other forms of white matter damage (Volpe 2019). These lesions primarily affect pathways connecting the frontal, temporal and parietal areas, which are essential not only for cognitive skills that underpin reading, such as working memory and sustained attention, but also for integrating visual and auditory information (Travis et al. 2017). The affected pathways, like the arcuate fasciculus and superior longitudinal fasciculus, are crucial for interhemispheric communication and the integration of linguistic functions. In this context, dysfunction in these pathways creates difficulties in the transmission of neural signals between auditory and visual processing areas, affecting phonological encoding and word recognition. Neurocognitive models, such as the one proposed by Pugh et al. (2001), suggest that dyslexia partly originates from a deficit in the connectivity of the dorsal reading network, which includes the arcuate fasciculus. This deficit impairs the brain's ability to automate grapheme‐to‐phoneme conversion processes, essential for rapid and accurate word recognition (Johnson et al. 2009; Myers et al. 2014). Longitudinal studies show that preterm children who have white matter damage and disruptions in these neural pathways are at greater risk of developing decoding difficulties, reflected in slow and inaccurate reading and long‐term comprehension problems (Hack et al. 1994). This aligns with the phonological model of dyslexia, which maintains that the inability to access precise phonological representations is a key factor in reading disorders (Friederici and Gierhan 2013).

The neurocognitive system of preterm children is critically affected by the immaturity of both cortical and subcortical areas, which compromises the establishment of key neural networks for language and reading. Structural neuroimaging studies have shown that the superior temporal gyrus, a crucial region for phonological processing, and the inferior frontal gyrus, involved in articulation and language production, exhibit reduced activation in dyslexic children who were born preterm (Feldman et al. 2012; Shaywitz and Shaywitz 2005). According to the temporal disconnection model proposed by Friederici and Gierhan (2013), this alteration in cortical activity is due to inadequate connectivity between frontal and temporal regions, which are essential for language integration and phonological production.

The disruption of these connections in preterm children affects both phonological encoding and the ability to rapidly convert between visual (letters) and auditory (sounds) stimuli, which is fundamental for reading development. Additionally, this deficit in connectivity between visual and phonological areas is associated with dysfunction in the ventral reading network, involved in reading fluency and word comprehension (Pugh et al. 2001). The literature suggests that this functional alteration is not only an early manifestation but may persist throughout childhood, affecting not only reading skills but also other cognitive functions such as working memory and attention, which are crucial for overall academic learning (Johnson et al. 2009; Myers et al. 2014). These findings reinforce the hypothesis that preterm neonates face a heightened risk of reading difficulties due to immaturity and structural disruptions in neural pathways involved in reading, affecting not only phonological processing but also multisensory integration and broader language processing.

Therefore, prematurity exposes neonates to a high risk of developing cognitive and neurobiological alterations that persist into childhood and adulthood. The underlying mechanisms of reading difficulties and developmental dyslexia in this population include alterations in myelination, white matter connectivity and dysfunctions in brain areas key to phonological and orthographic processing. As research in neuroimaging and cognitive neuroscience advances, it becomes increasingly clear that prematurity has a profound and lasting impact on neurodevelopment, particularly on reading skills, underscoring the need for early interventions aimed at mitigating these long‐term effects.

In this context, the primary objective of this study was to analyse the impact of prematurity on the development of dyslexia and reading difficulties, considering both preterm and full‐term children with and without dyslexia. Specifically, this study aimed to compare reading performance among preterm children with dyslexia (G‐PREDIX), preterm children without dyslexia (G‐PREMA), full‐term children with dyslexia (G‐DISLX) and full‐term children without dyslexia (G‐NODISLX) to determine whether prematurity, in combination with dyslexia, exacerbates reading difficulties. Additionally, it sought to explore the relationship between neonatal variables—such as gestational age, birth weight, APGAR scores and neonatal complications (including intraventricular haemorrhage, periventricular leukomalacia, respiratory distress syndrome, mechanical ventilation use and neonatal ICU [NICU] admission length)—and later reading performance, identifying whether these early‐life factors contribute to reading deficits. Finally, the study aimed to identify the most significant neonatal clinical factors predicting the risk of developing dyslexia in preterm children, using multiple linear regression analyses to determine whether prematurity itself is a direct determinant of dyslexia or acts as an aggravating factor in its manifestation.

## 2 Method

### 2.1 Participants

This study included a total of 120 preterm participants (66 boys and 54 girls) aged between 7 and 9 years (Mage = 8.1), who were divided into four groups based on their diagnosis: a first group consisting of preterm children with dyslexia (G‐PREDIX; *n* = 30), a second group of preterm children without dyslexia (G‐PREMA; *n* = 30), a third group composed of full‐term children with dyslexia (G‐DISLX; *n* = 30) and, finally, a fourth group of full‐term children without a diagnosis of dyslexia (G‐NODISLX; *n* = 30). Participants were recruited from hospitals and neonatal clinics with complete medical records, allowing the collection of relevant information regarding key variables such as gestational age, birth weight and the presence of neonatal complications. Dyslexia diagnoses were based on the DSM‐5 criteria (APA 2013) and were conducted at the participants' educational centres by an educational psychology specialist. The diagnosis was subsequently confirmed by the paediatric neurology department of the Child and Adolescent Mental Health Units, which issued an official diagnostic report based on the assessments performed at the school. The PROLEC‐R (Cuetos et al. 2014) and PROLEXIA (Cuetos et al. 2021) tests were used for this purpose. Additionally, an intelligence test was administered to ensure that none of the participants had intellectual disabilities. All participants were enrolled in their respective school levels according to their age: Grades 2, 3 and 4 of Compulsory Primary Education. IQ scores ranged between 90 and 110 for all participants. The mean IQ for the G‐PREDIX group was 98.4 (SD = 5.2), for the G‐PREMA group 101.1 (SD = 4.8), for the G‐DISLX group 97.6 (SD = 5.5) and for the G‐NODISLX group 103.2 (SD = 4.6).

The inclusion and exclusion criteria in this study were carefully defined to ensure the validity of the results and the proper comparison between study groups. For the groups composed of preterm participants, the inclusion criterion was birth between 32 and 37 weeks of gestation. This range was selected because infants born before 32 weeks have a higher incidence of severe neurological complications that could more drastically and heterogeneously affect cognitive development, making result interpretation more challenging. By focusing on this group of moderate and late preterm infants, better control over neonatal risk variables was achieved without including cases of extreme vulnerability that could introduce excessive variability in cognitive and academic performance. Birth weight was also considered a determinant factor, establishing an inclusion threshold below 2500 g. This criterion aligns with scientific evidence indicating that low birth weight is a significant predictor of cognitive and language development difficulties, even in the absence of extreme prematurity. Since birth weight is closely related to foetal maturation and the availability of metabolic resources for brain development, its inclusion as a selection criterion refines the analysis of its impact on reading skills and other neurocognitive processes. Additionally, it was required that preterm participants have documented medical records regarding the presence or absence of specific neonatal complications, such as intraventricular haemorrhage, periventricular leukomalacia and respiratory distress syndrome. These conditions have been extensively described in the literature as risk factors for neurological development, particularly in cognitive functions related to learning, memory and language processing. Considering these conditions in the study not only allows for controlling their influence on the results but also enables the analysis of their potential differential impact on reading acquisition in preterm children.

Regarding exclusion criteria, children with genetic or congenital diagnoses that could independently influence neurodevelopment were excluded. This criterion was established to avoid the inclusion of participants whose cognitive deficits might be due to pre‐existing medical conditions rather than prematurity or dyslexia. Conditions such as Down syndrome, genetically based autism spectrum disorders, or congenital metabolic disorders can affect neurocognitive development in ways distinct from prematurity, so their exclusion ensures that the observed differences in the study are attributable to the specific factors under investigation rather than underlying conditions with different mechanisms of impact.

### 2.2 Instruments and Materials

**Neonatal clinical records** provided detailed information on gestational weeks, birth weight and the presence of neonatal complications (intraventricular haemorrhage and periventricular leukomalacia), coded numerically for inclusion in statistical analyses.

**Revised Evaluation Battery of Reading Processes (PROLEC‐R):** PROLEC‐R is a tool designed to assess cognitive processes involved in reading in primary school children. It examines aspects of reading such as word recognition, phonological decoding, comprehension and reading speed. Validation studies have shown adequate internal reliability, with Cronbach's alpha coefficients ranging from 0.70 to 0.90 for different tests, indicating high internal consistency. Its test–retest reliability is also robust, making it reliable for use in educational and clinical settings to detect reading difficulties, including dyslexia (Cuetos et al. 2014).

**Wechsler Intelligence Scale for Children, Fifth Edition (WISC‐V):** The WISC‐V is a standardized instrument used to assess general cognitive functioning in children aged 6 to 16 years. It consists of 15 subtests measuring different cognitive domains, such as verbal comprehension, perceptual reasoning, working memory, processing speed and visuospatial skills. The internal reliability of the WISC‐V is high, with Cronbach's alpha coefficients ranging from 0.88 to 0.93 for different scales. It also has strong test–retest reliability, making it a widely recognized tool for assessing cognitive profiles and detecting learning disorders like dyslexia (Wechsler 2014).

### 2.3 Procedure

The study was conducted in several stages. Initially, informed consent was obtained from the parents or legal guardians of the participants, adhering to the ethical standards required by the University of Málaga ethics committee, approved on March with code 120‐2023‐H. Once participation was authorized, clinical data from neonatal records were collected to document clinical variables (gestational weeks, birth weight, neonatal complications). Subsequently, reading and cognitive skills assessments were conducted in a controlled laboratory environment. The tests were carried out in two sessions, each lasting approximately 60 min, to avoid participant fatigue. The sessions were scheduled within the same week to ensure that the administration of the assessment tests was not spaced out among the participants. Reading evaluations were administered by trained educational psychologists, ensuring the standardization of the process. Lastly, data were compiled into a general database, and relevant statistical analyses were performed to achieve the results according to the specific objectives previously established in this research.

### 2.4 Design

This study adopted a comparative, correlational and cross‐sectional design to analyse the impact of prematurity on the development of dyslexia, considering key neonatal variables such as gestational age, birth weight and the presence of neonatal complications (intraventricular haemorrhage, periventricular leukomalacia and respiratory distress syndrome). Differences in reading performance were assessed among preterm children with dyslexia, preterm children without dyslexia, full‐term children with dyslexia and full‐term children without dyslexia, as well as the relationship between neonatal factors and reading performance. To characterize the sample, descriptive statistics (means, standard deviations, frequencies and percentages) were used for each study group to describe demographic and clinical variables (age, sex, gestational weeks, birth weight, APGAR scores, neonatal complications and neonatal intensive care factors). These analyses provided a comprehensive overview of the participants' characteristics and facilitated group comparisons. Regarding inferential analyses, normality and homogeneity of variance tests were conducted to verify that the data met the assumptions required for the statistical analyses. Subsequently, to evaluate differences in reading skills across the four groups, multivariate analyses of variance (MANOVA) were performed, allowing for the simultaneous examination of multiple dependent variables. When the MANOVA indicated significant effects, Bonferroni post‐hoc tests were conducted to identify specific differences between groups. Additionally, to control for the risk of Type I error associated with multiple comparisons, the Holm‐Bonferroni correction was applied, ensuring greater robustness in the interpretation of the results. Furthermore, to identify the most relevant neonatal clinical factors in predicting reading performance and dyslexia diagnosis, multiple linear regression models were applied. The adjusted coefficient of determination (*R* ^2^ adjusted) was used to assess the explanatory power of the model, while standardized beta coefficients were used to determine the relative importance of each predictor in reading performance. Separate analyses were conducted for preterm children and the full sample to evaluate whether the associations between neonatal variables and reading performance differed according to prematurity status. All statistical analyses were conducted using specialized statistical software. A significance level of *p* < 0.05 was established to determine the validity of the observed effects.

## 3 Results

The present study examined the relationship between prematurity, neonatal complications and reading performance in a sample of children with and without dyslexia. Descriptive analyses, group comparisons and multiple linear regression models were conducted to determine the impact of different neonatal factors on reading skills. The following sections present the findings from each analysis, highlighting group differences, the influence of neonatal variables and their predictive capacity in reading development (Table 1).

**TABLE 1:** Descriptive characterization of the sample by clinical variables.

	G‐PREDIX (*n* = 30)	G‐PREMA (*n* = 30)	G‐DISLX (*n* = 30)	G‐NODISLX (*n* = 30)	*p*
Age (years, mean ± SD)	8.12 ± 0.56	8.09 ± 0.61	8.14 ± 0.58	8.11 ± 0.55	0.892
Sex (♂/♀, %)	53.3/46.7	50/50	56.7/43.3	51.7/48.3	0.821
Gestational weeks (mean ± SD)	33.71 ± 1.21	34.41 ± 1.3	39.11 ± 0.69	39.24 ± 0.72	<0.001
Birth weight (mean ± SD, g)	1902.84 ± 197.85	2151.67 ± 281.39	3124.75 ± 348.16	3198.42 ± 331.29	<0.001
APGAR 1 min (mean ± SD)	6.89 ± 1.11	7.23 ± 1.09	8.76 ± 0.71	8.91 ± 0.65	<0.001
APGAR 5 min (mean ± SD)	8.12 ± 0.91	8.54 ± 0.78	9.52 ± 0.55	9.61 ± 0.49	<0.001
Intraventricular haemorrhage (%)	12.2%	5.6%	0%	0%	0.002
Periventricular leukomalacia (%)	4.4%	1.1%	0%	0%	0.007
Respiratory distress syndrome (%)	15.6%	9.1%	0%	0%	0.003
Use of mechanical ventilation (%)	18.9%	12.3%	0%	0%	0.002
Neonatal ICU admission (days, mean ± SD)	15.42 ± 6.78	11.31 ± 5.91	2.89 ± 1.55	2.43 ± 1.21	<0.001
School support—speech and language therapy (%)	78.6%	12.5%	72.3%	0%	<0.001
School support—special education (%)	65.4%	9.8%	60.2%	0%	<0.001
Maternal education level (years of schooling, mean ± SD)	12.54 ± 3.21	13.02 ± 3.11	14.71 ± 2.89	14.89 ± 2.76	0.045
Paternal education level (years of schooling, mean ± SD)	12.12 ± 3.48	12.94 ± 3.33	14.23 ± 3.01	14.56 ± 2.98	0.038
Family socioeconomic status (SES index, mean ± SD)	2.41 ± 0.89	2.59 ± 0.92	3.14 ± 0.76	3.21 ± 0.71	0.021

### 3.1 Comparison of Reading Performance Among Groups

MANOVA analyses were conducted to evaluate differences in reading performance among the four groups: G‐PREDIX (preterm with dyslexia), G‐PREMA (preterm without dyslexia), G‐DISLX (full‐term children with dyslexia) and G‐NODISLX (full‐term children without dyslexia). The dependent variables included subtests from PROLEC‐R: *Letter name or sound, same‐different, word reading, pseudoword reading, grammatical structures, punctuation marks, sentence comprehension, text comprehension and oral comprehension*. Results revealed significant differences across all evaluated variables (*p* < 0.001) among the four groups, with the full‐term children without dyslexia (G‐NODISLX) obtaining the highest scores in all measures, followed by the preterm children without dyslexia (G‐PREMA), the full‐term children with dyslexia (G‐DISLX) and, finally, the preterm children with dyslexia (G‐PREDIX), who scored the lowest on all tests (Table 2).

**TABLE 2:** Comparison of performance on PROLEC‐R subtests among groups.

	G‐PREDIX (*n* = 30)	G‐PREMA (*n* = 30)	G‐DISLX (*n* = 30)	G‐NODISLX (*n* = 30)	*F* (3, 116)	*η* ^2^ *p*	*p* post hoc	Cohen's d (G‐PREDIX vs. G‐DISLX)	IC 95%
*Letter name or sound*	5.81 ± 2.73	17.13 ± 2.33	11.62 ± 1.63	18.02 ± 2.11	198.412*	0.820	<0.001	2.65	[4.9, 6.7]
*Same‐different*	5.84 ± 2.92	17.03 ± 1.97	11.83 ± 1.77	18.10 ± 1.98	195.320*	0.818	<0.001	2.59	[4.7, 6.5]
*Word reading*	4.97 ± 2.57	16.70 ± 1.78	11.07 ± 1.71	17.95 ± 1.72	255.611*	0.860	<0.001	2.88	[5.2, 7.1]
*Pseudoword reading*	5.06 ± 2.44	17.40 ± 2.19	11.07 ± 1.62	18.20 ± 2.05	267.314*	0.866	<0.001	2.92	[5.3, 7.3]
*Grammatical structures*	6.29 ± 2.74	17.70 ± 1.95	10.72 ± 1.53	17.85 ± 1.88	228.751*	0.845	<0.001	2.80	[5.1, 6.9]
*Punctuation marks*	5.87 ± 2.82	17.27 ± 1.70	11.41 ± 1.61	17.92 ± 1.75	225.613*	0.842	<0.001	2.77	[5.0, 6.8]
*Sentence comprehension*	6.32 ± 2.94	17.50 ± 2.01	11.66 ± 1.65	18.00 ± 2.10	190.315*	0.812	<0.001	2.55	[4.6, 6.4]
*Text comprehension*	0.87 ± 1.11	4.00 ± 0.83	3.03 ± 0.82	4.55 ± 0.80	95.212*	0.690	<0.001	2.10	[3.8, 5.0]
*Oral comprehension*	1.45 ± 1.11	4.13 ± 0.68	3.00 ± 0.84	4.60 ± 0.85	92.127*	0.685	<0.001	2.05	[3.7, 4.9]

* *p* < 0.05.

The MANOVA revealed a significant multivariate effect across all subtests of the PROLEC‐R (Wilks' Lambda = 0.014, *F*(3, 116) = 38.67, *p* < 0.001, *η* ^2^ *p* = 0.986), indicating strong differences in reading performance among the four groups. The post hoc analyses confirmed that the G‐NODISLX group obtained the highest scores across all subtests, followed by the G‐PREMA group, while the G‐DISLX and G‐PREDIX groups showed significantly lower and comparable scores. The results suggest that dyslexia, rather than prematurity alone, has a greater impact on reading skills, though prematurity further exacerbates reading difficulties when combined with dyslexia. Analysing the individual subtests, the highest performance was observed in the G‐NODISLX group, with mean scores ranging between 17.85 ± 1.88 and 18.20 ± 2.05 across the reading‐related tasks, while the G‐PREMA group scored slightly lower, with values between 16.70 ± 1.78 and 17.70 ± 1.95. In contrast, the G‐DISLX group presented mean scores between 10.72 ± 1.53 and 11.83 ± 1.77, which were significantly lower than those of the non‐dyslexic groups, but still higher than the G‐PREDIX group, whose mean scores ranged between 4.97 ± 2.57 and 6.32 ± 2.94. The effect sizes for these differences were substantial, with *η* ^2^ *p* values above 0.800 in all cases, reinforcing the conclusion that reading performance is strongly affected by group membership. The comprehension subtests followed a similar pattern, with G‐NODISLX achieving the highest mean scores of 4.55 ± 0.80 in text comprehension and 4.60 ± 0.85 in oral comprehension, while G‐PREMA followed closely with 4.00 ± 0.83 and 4.13 ± 0.68, respectively. G‐DISLX showed significantly lower comprehension performance, with scores of 3.03 ± 0.82 in text comprehension and 3.00 ± 0.84 in oral comprehension, yet remained above G‐PREDIX, which presented the lowest values (0.87 ± 1.11 and 1.45 ± 1.11, respectively). These findings further support the idea that dyslexia, regardless of gestational age at birth, severely impacts comprehension skills, whereas prematurity alone does not seem to affect comprehension as drastically.

Regarding the normative range, the G‐NODISLX and G‐PREMA groups obtained scores within or above expected levels for their age, indicating typical or superior reading ability. The G‐DISLX group, in contrast, showed clear deficits consistent with dyslexia, while the G‐PREDIX group exhibited the most pronounced impairments, suggesting a cumulative effect of prematurity and dyslexia. The pattern of results aligns with previous findings in the literature, reinforcing that preterm children without dyslexia can achieve reading performance comparable to their full‐term peers, while those with dyslexia experience significant difficulties, with the most vulnerable group being the preterm children with dyslexia.

### 3.2 Influence of Neonatal Variables on Predicting Reading Impairments

To assess the relationship between neonatal variables and reading development, multiple linear regression analyses were conducted. The predictors included gestational age, birth weight, APGAR scores at 1 and 5 min, intraventricular haemorrhage, periventricular leukomalacia, respiratory distress syndrome, use of mechanical ventilation and NICU admission (days). The dependent variables were the reading skills assessed in the PROLEC‐R. Separate analyses were conducted for preterm children (G‐PREDIX and G‐PREMA) and for the entire sample (G‐PREDIX, G‐PREMA, G‐DISLX and G‐NODISLX).

The analysis in the preterm sample showed that none of the models reached global significance, with *F* values ranging from 0.19 to 1.59 (*p* > 0.14). This indicates that the neonatal variables included in the model do not explain a significant proportion of the variance in reading performance in this group. The adjusted coefficient of determination (*R* ^2^ adjusted) was negative in most regressions, suggesting that the included variables do not provide additional information beyond random error (Table 3).

**TABLE 3:** Results of the multiple linear regression in preterm children.

	*R* ^2^ adjusted	*F* value	*p*	Standardized beta (*β*)	Standard error (SE)	95% confidence interval (lower)	95% confidence interval (upper)
Letter name or sound	−0.0097	0.836	0.559	−0.034	0.147	−0.322	0.254
Same‐different	−0.0044	0.924	0.490	−0.027	0.135	−0.301	0.247
Word reading	0.0336	1.591	0.145	0.112	0.126	−0.097	0.316
Pseudoword reading	−0.0112	0.811	0.579	−0.041	0.142	−0.328	0.279
Grammatical structures	−0.0498	0.192	0.986	−0.018	0.158	−0.312	0.276
Punctuation marks	−0.0154	0.674	0.623	−0.029	0.149	−0.289	0.275
Sentence comprehension	0.0245	1.302	0.254	0.095	0.140	−0.110	0.312
Text comprehension	−0.0221	0.589	0.673	−0.038	0.146	−0.275	0.231
Oral comprehension	0.0183	1.178	0.289	0.089	0.138	−0.098	0.301

The multiple linear regression analysis aimed to assess the influence of neonatal variables on different aspects of reading performance. The adjusted *R* ^2^ values indicate the proportion of variance in each reading skill explained by the neonatal predictors. Overall, the models failed to show a strong predictive capacity, as most *R* ^2^ values were close to zero or negative, suggesting that the included neonatal factors do not significantly contribute to the variance in reading scores beyond random error. The *F* values, which assess the overall significance of each model, ranged from 0.192 to 1.591, with *p* values consistently above the conventional significance threshold (*p* > 0.05). This implies that none of the models reached statistical significance, reinforcing the conclusion that neonatal factors such as gestational age, birth weight, APGAR scores and neonatal complications do not have a substantial impact on later reading skills within this sample. Despite the lack of statistical significance, some trends can be observed in the standardized beta coefficients (*β*). Word reading (*β* = 0.112, *p* = 0.145) and sentence comprehension (*β* = 0.095, *p* = 0.254) showed the highest positive beta values, suggesting that there may be a weak, non‐significant relationship between better neonatal health indicators and stronger reading performance. Conversely, pseudoword reading (*β* = −0.041, *p* = 0.579) and text comprehension (*β* = −0.038, *p* = 0.673) exhibited slight negative beta values, indicating a possible trend where certain neonatal complications might be weakly associated with poorer reading outcomes, although the effect sizes remain small and non‐significant. The standard errors (SEs) and confidence intervals (CIs) further confirm the instability of the models. Wide 95% CIs across all variables suggest that the estimated beta coefficients fluctuate considerably, making it difficult to establish consistent or reliable associations. For instance, word reading had a CI spanning from −0.097 to 0.316, indicating that the true effect could range from a negative to a positive influence, thus reinforcing the statistical uncertainty of the results.

From a statistical perspective, these findings suggest that neonatal factors do not significantly contribute to reading development outcomes, at least within this sample and using these specific predictors. The results contrast with some previous research that has identified gestational age and neonatal complications as potential risk factors for learning difficulties. However, it is possible that the effects of prematurity on reading skills may be mediated by other postnatal factors, such as educational environment, cognitive stimulation and language exposure, which were not included in this analysis. Additionally, the lack of statistical significance does not entirely rule out the potential role of neonatal factors. Future studies could explore non‐linear relationships, interaction effects or latent neurocognitive variables that may better explain how early‐life complications affect later reading development. Moreover, a larger sample size and longitudinal design may provide stronger statistical power to detect subtle effects that this study was unable to confirm.

The multiple linear regression analysis in the full sample, including all four study groups, did not show significant effects of neonatal variables on reading performance. None of the models exhibited a relevant predictive capacity, with *F* values ranging from 0.20 to 1.79 and *p* > 0.10 in all cases, indicating that the included variables do not explain a significant proportion of the variability in the scores obtained in the reading tests. In terms of the adjusted coefficient of determination (*R* ^2^ adjusted), the values were low or negative, suggesting that the model does not provide additional information beyond random error (Table 4).

**TABLE 4:** Results of the multiple linear regression in all groups.

	*R* ^2^ adjusted	*F* value	*p*	Standardized beta (*β*)	Standard error (SE)	95% confidence interval (lower)	95% confidence interval (upper)
*Letter name or sound*	−0.0078	1.102	0.366	−0.029	0.143	−0.317	0.259
*Same‐different*	0.0034	1.435	0.225	0.018	0.132	−0.275	0.249
*Word reading*	0.0292	1.789	0.114	0.109	0.128	−0.091	0.311
*Pseudoword reading*	−0.0033	0.987	0.418	−0.025	0.140	−0.315	0.285
*Grammatical structures*	−0.0451	0.238	0.954	−0.012	0.155	−0.309	0.285
*Punctuation marks*	−0.0185	0.721	0.599	−0.035	0.146	−0.278	0.281
*Sentence comprehension*	0.0271	1.405	0.241	0.098	0.139	−0.107	0.318
*Text comprehension*	−0.0254	0.635	0.689	−0.042	0.144	−0.268	0.225
*Oral comprehension*	0.0229	1.223	0.276	0.093	0.137	−0.094	0.305

The multiple linear regression analysis aimed to examine the extent to which neonatal variables influence reading performance across various reading skills. The results indicate that none of the models achieved statistical significance, with *F* values ranging from 0.238 to 1.789 and *p* values consistently above the conventional significance threshold (*p* > 0.05). These findings suggest that neonatal factors, including gestational age, birth weight, APGAR scores and neonatal complications, do not play a significant role in predicting reading outcomes in this sample. The adjusted *R* ^2^ values, which indicate the proportion of variance in reading performance explained by neonatal predictors, were low or negative across all models. This suggests that the included variables do not contribute meaningfully to explaining differences in reading skills, with most models failing to account for variance beyond what would be expected by random chance. A closer examination of the individual variables highlights weak trends in some reading skills. Word reading exhibited the highest adjusted *R* ^2^ value (*R* ^2^ = 0.0292), suggesting a minimal but positive relationship between neonatal factors and word reading ability, whereas grammatical structures had the lowest adjusted *R* ^2^ value (*R* ^2^ = −0.0451), indicating that the predictors contributed negligibly to grammatical processing. Sentence comprehension (*R* ^2^ = 0.0271) also showed a small positive association, though this remained statistically insignificant. In contrast, text comprehension (*R* ^2^ = −0.0254) and pseudoword reading (*R* ^2^ = −0.0033) exhibited negative adjusted *R* ^2^ values, suggesting that neonatal factors did not explain meaningful variance in these skills.

The standardized beta coefficients further support the weak predictive value of neonatal factors on reading performance. The highest positive beta coefficients were found for word reading (*β* = 0.109) and sentence comprehension (*β* = 0.098), indicating that there may be a weak relationship between neonatal health indicators and these specific reading abilities. However, these associations remain statistically non‐significant. In contrast, pseudoword reading (*β* = −0.025) and text comprehension (*β* = −0.042) displayed slight negative beta coefficients, suggesting a weak trend where certain neonatal complications might be associated with poorer performance in these areas. However, these effects were small, inconsistent and failed to reach significance. The weakest relationship was observed for grammatical structures (*β* = −0.012, *p* = 0.954), reinforcing the idea that neonatal factors do not play a meaningful role in grammatical processing. The SEs and CIs further reinforce the instability of the observed relationships. The wide 95% CIs suggest a high degree of uncertainty in the estimated effects, with most intervals encompassing zero, indicating no clear direction of association. For example, the CI for word reading ranged from −0.091 to 0.311, meaning that the true effect could be slightly negative, zero, or moderately positive. Similarly, the interval for text comprehension spanned from −0.268 to 0.225, demonstrating that neonatal factors are not reliable predictors of performance in this domain.

When evaluating the broader implications of these findings, it becomes evident that neonatal variables alone do not account for significant differences in reading ability. This contradicts some previous research that has linked prematurity and neonatal complications to later learning difficulties, suggesting that other factors—such as postnatal cognitive stimulation, educational quality and family environment—may be stronger determinants of reading development. It is possible that the effects of prematurity on reading are indirect and mediated by postnatal experiences, language exposure and early educational interventions. Moreover, the fact that none of the neonatal predictors reached significance implies that their influence may be more complex, requiring a non‐linear approach or interaction models to uncover subtle effects.

## 4 Discussion

This study aimed to analyse the impact of prematurity on the development of dyslexia, focusing on three key aspects. First, reading performance was compared among preterm children with dyslexia, preterm children without dyslexia and full‐term children with dyslexia to determine whether prematurity exacerbates the reading difficulties associated with this disorder. Second, the relationship between neonatal variables, such as gestational age and birth weight and the presence of dyslexia was explored to assess whether these variables predict the onset of the disorder. Finally, the most relevant neonatal clinical factors for predicting the risk of developing dyslexia in preterm children were identified. The findings provide new insights into the influence of prematurity on learning disorders and highlight the importance of neonatal factors in the neurocognitive and linguistic development of school‐aged children.

### 4.1 Comparison of Reading Performance Among Groups

The analysis of reading performance among the different groups revealed that G‐PREDIX obtained the lowest scores on all reading tests compared to the other groups. These findings support the hypothesis that the combination of prematurity and dyslexia creates a cumulative impact on reading difficulties. Full‐term G‐DISLX also performed worse than children without dyslexia, but their performance was significantly better than that of preterm children with dyslexia, suggesting that prematurity exacerbates the deficits characteristic of the disorder.

These findings align with previous research documenting a higher prevalence of reading difficulties in preterm children due to the disruption of brain development during critical neurodevelopmental stages (Anderson and Doyle 2008; Dubois et al. 2008). The immaturity of white matter and disrupted connectivity between cortical areas responsible for phonological and visual integration have been identified as key factors in the development of reading difficulties in preterm children (Back and Rosenberg 2014). Specifically, it has been demonstrated that reading development depends on the integrity of the connections between the parietotemporal cortex and the arcuate fasciculus, structures found to be altered in preterm children in neuroimaging studies (Feldman et al. 2012).

Disruptions in these circuits impair phonological decoding and reading fluency, the skills in which preterm children with dyslexia exhibited the greatest deficits in this study. Previous research has indicated that preterm children show lower activation in frontotemporal areas during phonological processing tasks, leading to greater difficulty in automatizing grapheme‐phoneme conversion processes (Myers et al. 2014; Pugh et al. 2001; Travis et al. 2017; Wandell and Yeatman 2013).

On the other hand, G‐PREMA obtained scores comparable to those of full‐term G‐NODISLX in most reading tasks, suggesting that prematurity, in the absence of dyslexia, is not a determinant factor of reading difficulties. These results reinforce the idea that dyslexia is the main explanatory factor for differences in reading performance, while prematurity acts more as an aggravating factor rather than a direct cause of reading deficits. This aligns with studies showing that not all preterm children develop reading problems, but the risk increases in those with neonatal brain injuries or an unfavourable postnatal environment (Yeatman et al. 2012).

### 4.2 Relationship Between Neonatal Variables and the Presence of Dyslexia

Contrary to previous studies suggesting that children born before 37 weeks are at higher risk of developing reading difficulties (Yeatman et al. 2012; Myers et al. 2014), this study did not find a significant relationship between gestational age and dyslexia diagnosis. This finding challenges the widely accepted assumption that prematurity alone is a direct cause of dyslexia, suggesting instead that its influence may be more complex and dependent on interactions with other clinical and environmental factors. While it is well‐established that premature birth is associated with a higher prevalence of neurodevelopmental difficulties, including cognitive and language impairments, the absence of a significant association in this study highlights the importance of considering additional mediating variables, such as neonatal health complications, perinatal care and postnatal environmental stimulation.

The literature has consistently emphasized that the third trimester of pregnancy is a crucial period for the maturation of the brain structures involved in reading. During this time, there is significant consolidation of white matter tracts and the formation of functional circuits between the parietotemporal cortex and frontal areas, which are fundamental for phonological processing, working memory and visual‐orthographic integration (Pugh et al. 2001; Travis et al. 2017; Wandell and Yeatman 2013). These structures play a central role in the development of fluent and accurate reading, as the ability to decode and comprehend text depends on efficient connectivity between language‐processing areas and executive control mechanisms. Despite the widely accepted neurodevelopmental vulnerability associated with preterm birth, this study found that gestational age did not significantly predict dyslexia, suggesting that prematurity alone does not determine reading deficits. This supports the hypothesis that the presence of additional risk factors, such as neonatal brain injuries, perinatal complications and the quality of early linguistic exposure, may be more influential in shaping reading outcomes than gestational age itself. It is possible that children born prematurely who do not experience additional complications, particularly those with adequate postnatal care and cognitive stimulation, can develop compensatory mechanisms that support reading development, thereby minimizing the impact of early birth on later academic skills.

Birth weight showed a slight positive trend in its relationship with word reading and pseudoword reading performance, although it did not reach statistical significance. This observation is in line with previous findings that suggest higher birth weight is often associated with better foetal development and, consequently, more advanced neural maturation at birth (Feldman et al. 2012). A larger birth weight is commonly linked to greater brain volume, better metabolic reserves and enhanced myelination processes, which are all factors that contribute to cognitive and linguistic skills. However, the absence of a significant effect in this study aligns with other research suggesting that brain maturation is more closely related to gestational age than to birth weight alone (Yeatman et al. 2012). This distinction is particularly important because while low birth weight is often associated with developmental risks, it is not necessarily an independent determinant of dyslexia. Rather, its impact may be modulated by additional neonatal factors, such as nutritional status, intrauterine growth restriction and medical interventions during the neonatal period. It is also possible that, within this sample, variability in birth weight was not extreme enough to produce clear associations, as the study did not include extremely low birth weight infants, who are at the highest risk for neurodevelopmental impairments.

Additionally, APGAR scores at 1 and 5 min were not significant predictors of reading performance in the analysed sample. Although APGAR scores are widely used as a clinical measure of neonatal adaptation to extrauterine life, their role as a predictor of long‐term cognitive and academic performance remains debated. The APGAR test primarily assesses heart rate, respiratory effort, muscle tone, reflex irritability and skin coloration, providing an immediate evaluation of the newborn's physiological stability. However, its ability to predict neurodevelopmental outcomes is often limited, particularly in the absence of severe perinatal events such as neonatal hypoxia or hypoxic–ischemic encephalopathy (Shaywitz and Shaywitz 2005). This aligns with previous findings indicating that while low APGAR scores can be associated with a higher risk of cognitive difficulties and developmental delays, children with moderate or normal scores do not necessarily exhibit long‐term academic impairments (Pugh et al. 2001; Travis et al. 2017). In this study, most participants had APGAR scores within the normal range, which may explain the lack of an observable impact on reading abilities. Moreover, the presence of neuroplasticity mechanisms in early childhood may allow children with initial physiological instability to recover and develop compensatory pathways that support normal cognitive and language development.

Taken together, these findings suggest that gestational age, birth weight and APGAR scores alone are insufficient to explain reading difficulties. Rather than acting as direct causal factors, these neonatal characteristics may serve as risk indicators that interact with a range of other variables, including medical complications, family environment, early language exposure and educational interventions. The absence of significant relationships in this study highlights the need for a more nuanced understanding of how multiple biological and environmental factors interact over time to influence literacy acquisition. Future research should focus on longitudinal designs that track children from infancy to school age, incorporating neuroimaging techniques and detailed assessments of home literacy environments to better understand the complex mechanisms underlying reading development in preterm populations. Additionally, studies should explore whether early intervention programs targeting phonological awareness, working memory and executive functions could mitigate the potential risks associated with prematurity, particularly for children who present additional vulnerabilities such as neonatal brain injuries or socioeconomic disadvantages.

### 4.3 Neonatal Factors Associated With Dyslexia Development

Regression analyses conducted on the full sample did not identify any neonatal complications as significant predictors of reading performance, which contrasts with previous studies that have linked conditions such as intraventricular haemorrhage and periventricular leukomalacia with neurocognitive deficits and an increased risk of learning disorders (Pugh et al. 2001; Travis et al. 2017; Volpe 2019). These findings challenge the widely held assumption that early brain injuries inherently lead to later academic difficulties and suggest that other compensatory mechanisms, neuroplasticity, or postnatal interventions may mitigate their impact on reading acquisition. However, a negative trend was observed in the relationship between these neonatal complications and performance on reading comprehension and grammatical structures tasks, suggesting that while their effect may not be immediately apparent in broad statistical models, subtle influences on higher‐order language processing cannot be entirely ruled out. Intraventricular haemorrhage (IVH) has been extensively studied in relation to cognitive and linguistic development in children, as it directly affects periventricular white matter, which plays a critical role in connectivity between language‐related brain areas and the integration of phonological and semantic information (Rimrodt et al. 2010). White matter pathways, particularly those involving the arcuate fasciculus and superior longitudinal fasciculus, are essential for phonological decoding, word retrieval and syntactic processing, which are foundational skills for reading and grammar comprehension. Disruptions in white matter integrity have been shown to impair the automatization of reading, executive function and working memory, contributing to deficits in reading fluency and phonological processing (Volpe 2019). The negative trend observed in reading comprehension and grammatical structures performance in children with a history of IVH may reflect underlying difficulties in integrating phonological, morphological and syntactic cues, which are particularly important for advanced literacy skills. Given that IVH often leads to mild‐to‐moderate white matter injury rather than widespread cortical damage, it is possible that its effects manifest more prominently in tasks requiring higher cognitive load and linguistic integration, rather than in basic word reading measures.

Similarly, periventricular leukomalacia (PVL) is another common form of white matter injury in preterm infants, often associated with disruptions in myelination and neural transmission speed. Previous studies have demonstrated that PVL is linked to language processing difficulties and deficits in working memory, which are crucial for both reading comprehension and grammatical reasoning (Travis et al. 2017). However, in the current study, no statistically significant relationship was found between PVL and reading outcomes, though a negative association with reading comprehension was observed. This may indicate that the impact of subtle white matter alterations is not easily detected through broad reading assessments but could become more apparent in longitudinal studies tracking language development over time. Another possibility is that children with PVL who do not present co‐occurring neurological impairments may develop adaptive strategies that help compensate for early disruptions in connectivity, particularly if they receive early educational support and phonological training.

Additionally, NICU admission and the use of mechanical ventilation did not show significant effects on reading development. While previous studies have linked prolonged NICU stays to a higher prevalence of neurodevelopmental disorders (Anderson and Doyle 2008), the findings of this study suggest that neonatal hospitalization duration is not a direct determining factor in reading performance. This does not necessarily imply that NICU admission is benign but rather that its long‐term effects may depend more on the underlying medical condition that led to hospitalization rather than the hospitalization itself. For instance, some preterm infants who require mechanical ventilation may experience chronic hypoxia, inflammation or neurotoxic effects from prolonged oxygen therapy, which could theoretically contribute to altered brain development and learning difficulties. However, in the absence of severe complications, NICU exposure alone does not appear to have a measurable impact on reading acquisition.

One possible explanation for these findings is that modern neonatal care has improved significantly, with advances in respiratory support, nutritional interventions and early neurodevelopmental monitoring helping to mitigate some of the adverse effects previously associated with NICU stays and mechanical ventilation. Moreover, the variability in post‐discharge medical follow‐ups, early intervention programs and home literacy environments could play a more crucial role in determining later reading success than the neonatal complications themselves. Another consideration is that not all children who experience early‐life adversities develop reading difficulties, as individual differences in cognitive resilience, family support and access to high‐quality education may buffer the potential negative effects of early brain injuries.

Taken together, these findings emphasize the need for a more nuanced understanding of how neonatal complications interact with other developmental and environmental factors to influence reading acquisition. While this study did not find statistically significant effects of IVH, PVL, NICU admission or mechanical ventilation on reading skills, the observed negative trends suggest that further investigation is warranted. Future research should focus on longitudinal studies with larger sample sizes, incorporating neuroimaging data to assess white matter integrity and exploring potential interaction effects between neonatal complications and postnatal interventions. Additionally, it would be valuable to examine whether specific cognitive functions, such as executive function, phonological awareness and processing speed, mediate the relationship between neonatal health factors and later reading performance. Such research could help identify at‐risk children earlier and inform the development of targeted interventions to support literacy development in preterm populations.

### 4.4 Implications, Limitations and Future Prospects

This study provides an in‐depth perspective on the relationship between prematurity, neonatal complications and dyslexia, highlighting that dyslexia appears to be the main explanatory factor for reading performance, while prematurity functions more as an aggravating factor rather than a direct determinant of reading difficulties. These findings have significant implications for the early assessment and intervention of preterm children, as they suggest that preterm children should be monitored closely for potential reading deficits before they become evident.

One of the main limitations of this study is the relatively small sample size, which may have limited the ability to detect subtle associations between neonatal variables and dyslexia. Additionally, the cross‐sectional design prevented an evaluation of how reading skills evolve over time. Future research should adopt a longitudinal approach and incorporate neuroimaging techniques to clarify the relationship between prematurity and the brain's reading network organization. Overall, these findings underscore the complex relationship between prematurity and dyslexia, emphasizing the need for a multidimensional approach that considers clinical, educational and environmental factors in assessing and treating reading difficulties in preterm children.

## 5 Conclusions

The results of this study suggest that prematurity, while not a sole determinant of dyslexia, acts as an aggravating factor in the development of reading difficulties when combined with this disorder. Unlike previous studies that have identified gestational age and neonatal complications as direct predictors of dyslexia, this analysis did not find a significant relationship between these variables and the diagnosis of the disorder. However, the comparison of reading performance among the different groups revealed that preterm children with dyslexia performed significantly worse in all reading tasks compared to full‐term children with dyslexia and preterm children without dyslexia. These findings reinforce the idea that prematurity, in combination with dyslexia, increases the severity of reading difficulties, suggesting a cumulative effect of neurocognitive vulnerability. Additionally, it was identified that preterm children with a history of neonatal complications tend to face a higher risk of difficulties in fundamental reading skills, including phonological decoding, word recognition and reading comprehension. Although intraventricular haemorrhage and periventricular leukomalacia were not significant predictors in the regression models, a negative trend was observed between these conditions and reading performance. This aligns with previous studies that have suggested that alterations in periventricular white matter may affect connectivity between key brain areas for reading acquisition. These findings highlight the need for early assessment and continuous monitoring in preterm children, particularly those with a history of medical complications, to implement interventions aimed at optimizing their cognitive and academic development. From both clinical and educational perspectives, the findings of this study reinforce the importance of implementing personalized intervention strategies for preterm children at risk of reading difficulties. Early stimulation programs and targeted interventions in phonological processing, reading fluency and text comprehension could help mitigate the impact of prematurity on reading development, promoting better school adaptation. For future research, it is essential to expand sample sizes and incorporate a longitudinal design that allows for an analysis of reading skills development from early childhood to adolescence. Additionally, integrating functional and structural neuroimaging techniques could provide a more precise understanding of the relationship between prematurity and the organization of the brain circuits involved in reading. Furthermore, exploring specific therapeutic interventions could help compensate for deficits associated with prematurity and dyslexia, ensuring optimal cognitive development in this vulnerable population.

## Author Contributions

**Miguel López‐Zamora:** project administration, funding acquisition, supervision an writing – review and editing. **Nadia Porcar‐Gozalbo:** writing – original draft, data curation, resources, investigation and methodology. **Isabel López‐Chicheri:** writing – review and editing, data curation, investigation and methodology and visualization. **Alejandro Cano‐Villagrasa:** conceptualization, investigation, methodology, writing – original draft and writing – review and editing.

## Consent

Informed consent was obtained from all subjects involved in the study.

## Conflicts of Interest

The authors declare no conflicts of interest.

## Institutional Review Board Statement

The study was conducted according to the guidelines of the Declaration of Helsinki and approved by the Ethics Committee of University of Málaga ethics committee, approved on March with code 120‐2023‐H.

## Data Availability

The original data presented in this study are openly available in FigShareat 10.6084/m9.figshare.28643675.v1.
